# A Comparison of Statistical Methods for Studying Interactions of Chemical Mixtures

**DOI:** 10.1007/s12561-023-09415-4

**Published:** 2024-02-23

**Authors:** Debamita Kundu, Sungduk Kim, Mary H. Ward, Paul S. Albert

**Affiliations:** 1 Biostatistics Division, Public Health Sciences, School of Medicine, University of Virginia, Charlottesville, VA, USA; 2 Biostatistics Branch, Division of Cancer Epidemiology and Genetics, National Cancer Institute, Bethesda, MD, USA; 3 Occupational and Environmental Epidemiology Branch, Division of Cancer Epidemiology and Genetics, National Cancer Institute, Bethesda, MD, USA

**Keywords:** Bayesian kernel machine regression, Chemical mixture, Interaction, Latent class model, Shrinkage prior

## Abstract

Properly assessing the effects of environmental chemical exposures on disease risk remains a challenging problem in environmental epidemiology. Various analytic approaches have been proposed, but there are few papers that have compared the performance of different statistical methods on a single dataset. In this paper, we compare different regression-based approaches for estimating interactions between chemical mixture components using data from a case-control study on non-Hodgkin’s lymphoma. An analytic challenge is the high percentage of exposures that are below the limit of detection (LOD). Using imputation for LOD, we compare different Bayesian shrinkage prior approaches including an approach that incorporates the hierarchical principle where interactions are only included when main effects exist. Further, we develop an approach where main and interactive effects are represented by a series of distinct latent functions. We also fit the Bayesian kernel machine regression to these data. All of these approaches show little evidence of an interaction among the chemical mixtures when measurements below the LOD were imputed. The imputation approach makes very strong assumptions about the relationship between exposure and disease risk for measurements below the LOD. As an alternative, we show the results of an analysis where we model the exposure relationship with two parameters per mixture component; one characterizing the effect of being below the LOD and the other being a linear effect above the LOD. In this later analysis, we identify numerous strong interactions that were not identified in the analyses with imputation. This case study demonstrated the importance of developing new approaches for mixtures when the proportions of exposure measurements below the LOD are high.

## 1 Introduction

In environmental epidemiology, interest often focuses on estimating the complex associations between environmental chemical mixtures and disease risk. Recently, various approaches have focused on characterizing higher-order interactions between mixture components and outcomes including regression-based [1, 2], machine kernel regression [3], and latent class modeling approaches [4–6]. Although these methods have been illustrated with actual chemical mixture data, there have been few papers that have compared the various approaches on an actual dataset. This article investigates the different modeling strategies using case–control study data examining the effects of chemical exposures on non-Hodgkin’s Lymphoma (NHL).

There are analytic issues that make comparisons interesting. First, exposures can be non-linear making inferences about interactions more complex. Second, many of the chemical exposure measurements were below the lower limit of detection (LOD). Third, some of the chemicals were highly correlated. A number of articles have focused on developing summary score measures that relate mixtures to disease outcomes. These methods focus on estimating a linear combination of the numerous mixture components and relating this combination to either a continuous or binary outcome [7, 8]. The focus of this article is on understanding the complex interactions between the mixture components, and we therefore compare methodologies where this is the goal.

We present the NHL case–control study in Sect. 2. In all subsequent sections, we describe the various methods followed by an analysis of these data using each of the approaches. In Sect. 3, we review the Bayesian kernel machine regression (BKMR). Section 4 presents the broad class of shrinkage prior regression-based approaches including the recent methodology that incorporates a hierarchical constraint for interaction estimation. We also examine a novel approach to account for LOD using a multi-parameter per exposure formulation. Section 5 extends a recently developed latent class formulation [5] to the interaction setting. Finally, in Sect. 6 we present a discussion of the results along with future next steps for methodological development.

## 2 NCI-SEER NHL Study

Studying the relationships between environmental and occupational exposure to chemicals and cancer risk remains an important area in cancer research (see IARC website). The NCI-SEER NHL study [9] is a population-based case–control study that was designed to determine the associations between chemical exposures (including pesticides and insecticides) found in used vacuum cleaner bags and the risk of NHL. Often chemicals enter the household from indoor use or drift in from outdoor and may persist for months and years in carpet and cushion furniture without being degraded by sunlight, rain, and extreme temperature. Hence, carpet dust sampling provides a more objective basis for exposure assessment as it contains integrated chemical exposure over a long period which is potentially more relevant to disease risk than recent or current exposure. In this study, the samples were collected from used vacuum cleaner bags of 672 cases and 508 control subjects in Detroit, Iowa, Los Angles, and Seattle and were analyzed for chemicals [9]. Primarily the laboratory measurements contain missing data due to concentrations being below the LOD. The median percent of observations below the detection limit was 61% (across chemicals) with a range of (3% to 93%). In study analyses, multiple imputation was performed to “fill-in” exposure measurements that were below the LOD. This imputation was done by assuming that chemicals were log-normally distributed and that values below the LOD were in the tails of the distribution. Particularly for chemicals with a high percentage of values below their detection limits, results may not be robust to misspecification of the parametric assumptions. Thus, we consider alternative less model-based approaches to account for LOD.

**Fig. 1:**
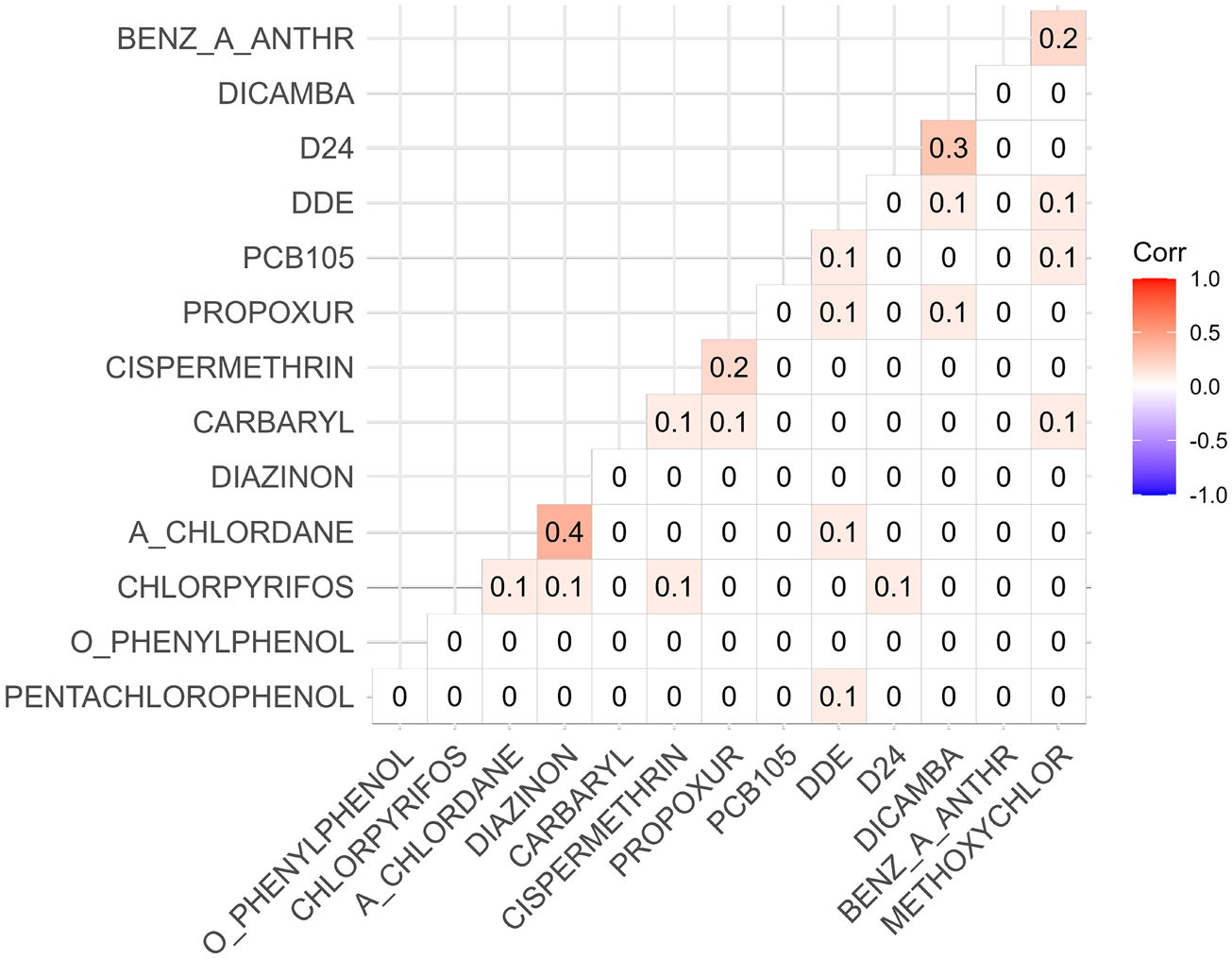
Correlation plot for chemical exposures

There were a few groups of chemicals where members within a group were highly correlated with each other (Correlation > 0.9). In this case, we randomly chose one member for each highly correlated pair in the analysis. Exposure data were logtransformed since measurements on the original scale were highly skewed. There were 26 chemicals exposures measured which are listed in the Appendix. After filtering out highly correlated chemicals, there was a total of 14 chemicals. Thus, the final dataset contained 14 chemical exposures on 1180 individuals (508 controls and 672 cases). Figure 1 shows the correlation between the 14 chemicals. We considered site, sex, education, and age as covariates [9] in all models for our data application.

## 3 Bayesian Kernel Machine Regression (BKMR)

**Fig. 2:**
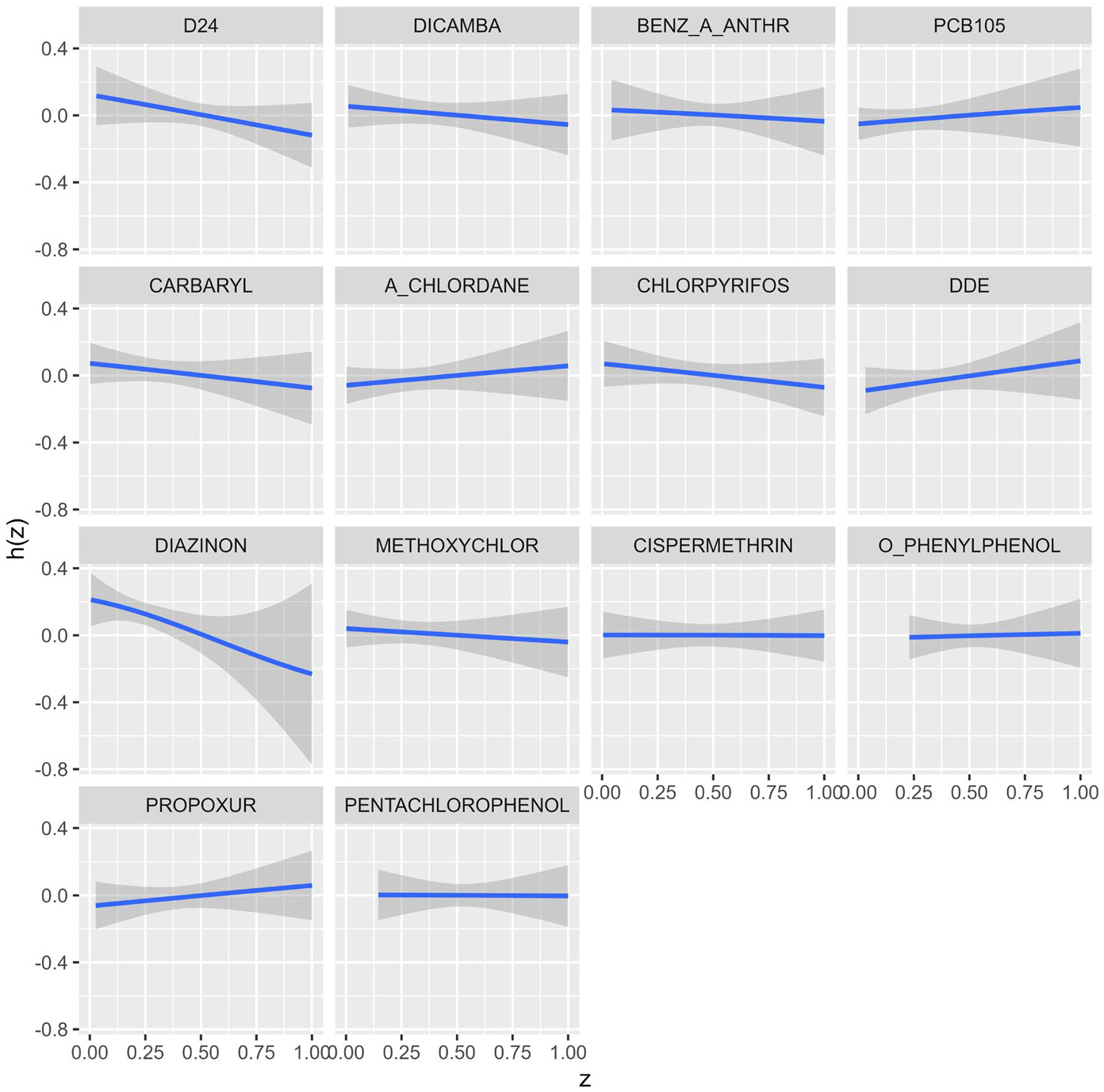
Univariate exposure–response estimation

A popular statistical method for analyzing chemical mixture data is the Bayesian kernel machine regression approach. In this approach, Bobb et al. [3] modeled non-linear and non-additive relationships between exposure variables and outcome through a non-parametric kernel function. For a binary outcome $Y_{i}$, the kernel machine regression is implemented through a probit link

(1) $$\Phi^{-1}\left(P\left(Y_{i}=1\right)\right)=h\left(X_{i1},X_{i2},\ldots ,X_{ip}\right)+U_{i}^{\prime }\alpha ,$$

where Φ denotes the cumulative distribution function (CDF) of the standard normal distribution, h(⋅) is the flexible function of p exposure variables $X_{i1},X_{i2},\ldots ,X_{ip}$, and α defines the vector of regression coefficients for covariates $U_{i}$. The function h(⋅) is characterized as a Gaussian kernel function, where $h={\left(h_{1},h_{2},..,h_{N}\right)}^{\prime }$ is multivariate normal with mean **0** and correlation given by $\text{cor}\left(h_{i},h_{i^{\prime }}\right)=\text{exp}\left(\tau \sum_{p=1}^{P}{\left(X_{ip}-X_{i^{\prime }p}\right)}^{2}\right)$ for all pairs of individuals i and $i^{\prime }$. Further, they model the latent variable $Y_{i}^{*}$ (in Eq. (1)) as

(2) $$Y_{i}^{*}=h\left(X_{i1},X_{i2},\ldots ,X_{ip}\right)+U_{i}^{\prime }\alpha +\epsilon_{i},\;i=1,\;2,\ldots ,N,$$

where $\epsilon_{i}\sim N(0,1)$. The formulation results in a probit link function when we dichotomize the latent variable at zero such that $Y_{i}=1$ when $Y_{i}^{*}>0$ and 0 otherwise. We used the *kmbayes* function from the BKMR package to fit the model on the NHL data. Figure 2 shows the univariate exposure–response relationships for NHL with each chemical when the remaining chemicals are fixed at their median values. The plot suggests none of the chemicals have a sizeable effect on cancer risk.

**Fig. 3:**
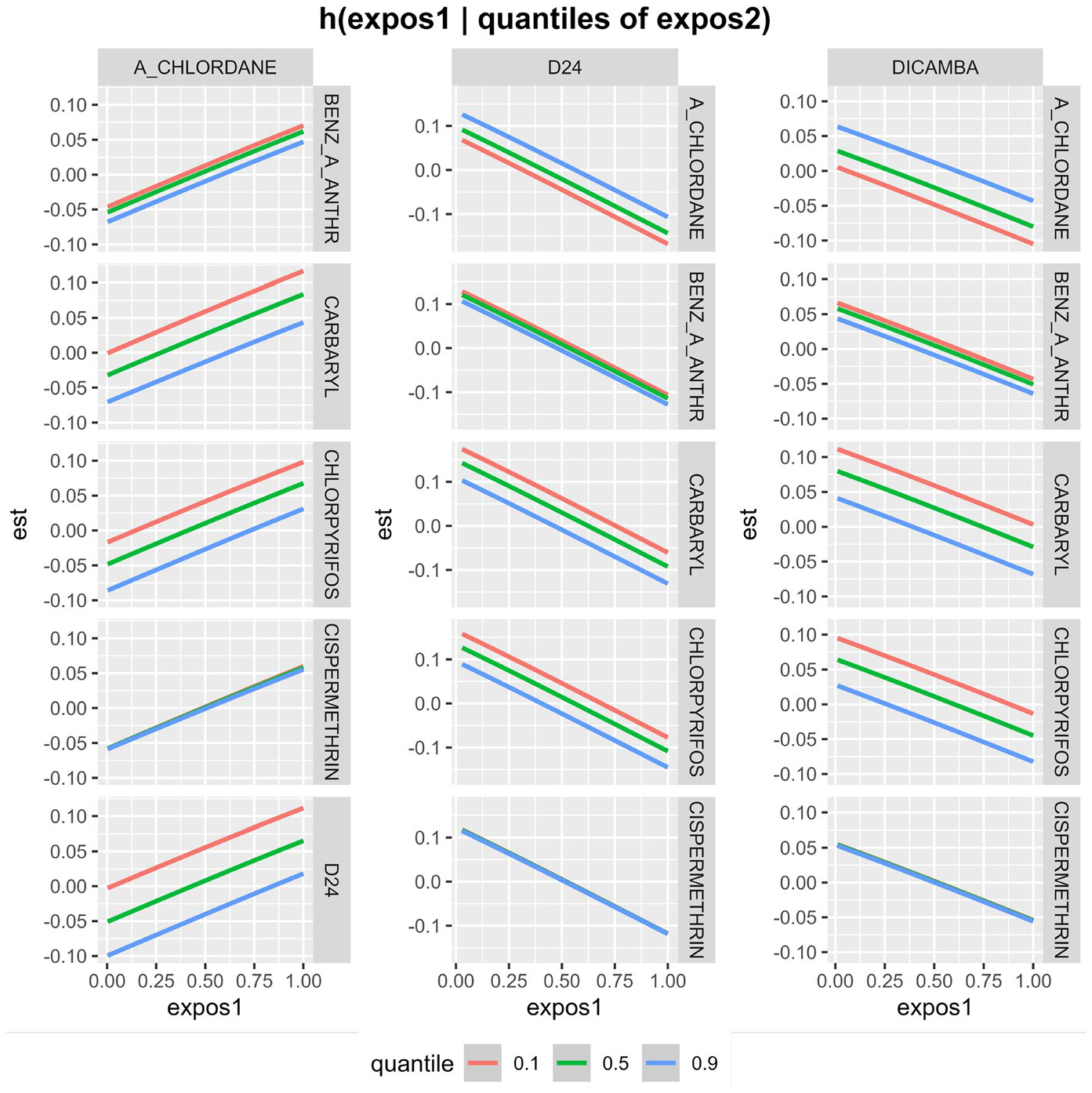
Bivariate exposure–response estimation

Two-way interactions among all pairs of exposures can be characterized by estimating the conditional distribution of the effect of one exposure given quantiles of the second exposure with the remaining chemicals fixed at their median value. Figure 3 shows the bi-variate exposure–response relationship derived from the BKMR analysis for a subset of pairwise comparisons. The fact that for each chemical, the conditional distributions are parallel for different quantiles of other chemicals suggests no evidence of interaction effects. We saw similar parallelism for all 91 interaction terms suggesting no interactions among the 14 chemicals (data not shown).

## 4 Bayesian Shrinkage Methods

Shrinkage priors in Bayesian estimation provide a useful way to estimate the higher-order interactions among mixture components. These approaches are analogs to penalized likelihood approaches that have been proposed in the frequentist context, and have the advantage in that they incorporate the penalization/shrinkage into the inference of the model parameters [10].

In this section, we compare various Bayesian shrinkage methods for estimating the interactions among components of chemical mixtures. We consider the following logistic regression model with linear effects consisting of p chemical exposures or main effects and p(p-1)/2 two-way interactions effects:

(3) $$\text{logit}P\left(Y_{i}=1|X_{i},U_{i}\right)=U_{i}^{'}\alpha^{\text{*}}+\overset{p}{\underset{j=1}{\sum }}X_{ij}\beta_{j}^{\text{*}}+\overset{p}{\underset{j=1}{\sum }}\overset{p-1}{\underset{k=j+1}{\sum }}X_{ij}X_{ik}\gamma_{jk}^{\text{*}},\;i=1,2,\ldots ,N,$$

where $Y={\left(Y_{1},Y_{2},\cdots ,Y_{N}\right)}^{\prime }$ denotes the binary health response for N individuals, $X_{i}={\left(X_{i1},X_{i2}\ldots ,X_{ip}\right)}^{\prime }$ denotes p-dimensional continuous vector of main effects. We also denote $\text{logit }a=\text{log}\frac{a}{1-a},U_{i}={\left(U_{i1},U_{i2}\ldots ,U_{iq}\right)}^{\prime }$ as q-dimensional covariate vector including the intercept term, $\alpha^{*}={\left(\alpha_{1},\alpha_{2},\ldots ,\alpha_{q}\right)}^{\prime }$ as the corresponding q-dimensional regression coefficient vector, $\beta_{j}^{*}$ as the main effect regression coefficient of the $j^{\text{th}}$ chemical, and $\gamma_{jk}^{*}$ as the interaction effect regression coefficient of the $j^{\text{th}}$ and $k^{\text{th}}$ chemicals.

Following a latent variable approach [11], we approximate Eq. (3) using a robit link [12]. Let $\xi ={\left(\xi_{1},\xi_{2},\ldots ,\xi_{N}\right)}^{\prime }$ be a N-dimensional latent vector such that $Y_{i}=1$, if $\xi_{i}>0$ and 0 otherwise, where $\xi_{i}=U_{i}^{\prime }\alpha^{*}+\sum_{j=1}^{p}X_{ij}\beta_{j}^{*}+\sum_{j=1}^{p}\sum_{k=j+1}^{p-1}X_{ij}X_{ik}\gamma_{jk}^{*}+\epsilon_{i}$. The robit link function, indexed by v, results if $\epsilon_{i}$ follows a student t-distribution with v degrees of freedom [13], i.e., $P\left(Y_{i}=1|\alpha^{*},\beta^{*},\gamma^{*}\right)=F_{t_{v}}\left(U_{i}^{\prime }\alpha^{*}+\sum_{j=1}^{p}X_{ij}\beta_{j}^{*}+\sum_{j=1}^{p}\sum_{k=j+1}^{p-1}X_{ij}X_{ik}\gamma_{jk}^{*}\right)\;$, where $\beta^{*}={\left(\beta_{1}^{*},\beta_{2}^{*},\ldots ,\beta_{p}^{*}\right)}^{\prime }$ and $\gamma^{*}={\left(\gamma_{11}^{*},\gamma_{12}^{*},\ldots ,\gamma_{p(p-1)/2}^{*}\right)}^{\prime }$. As v→∞, the *robit*(v) model becomes the probit regression model. Liu [12] suggested that the *robit* link with v=7 degrees of freedom closely approximates the *logit* link with $\alpha_{l}=\alpha_{j}^{*}/1.5484,\;\beta_{j}=\beta_{j}^{*}/1.5484$, and $\gamma_{jk}=\gamma_{jk}^{*}/1.5484$. Moreover, we use the fact that the t-distribution can be represented as a scale mixture of normal distribution by introducing a mixing variable $\lambda_{i}$, such that $\epsilon_{i}\left|\lambda_{i}\sim \text{N}\left(0,\frac{1}{\lambda_{i}}\right)\right.$ and $\lambda_{i}\sim \text{G}\left(\frac{v}{2},\frac{v}{2}\right)$, where $\text{N}\left(\mu ,\sigma^{2}\right)$ denotes a normal distribution with mean μ and variance $\sigma^{2}$ and $\text{G}\left(c_{1},c_{2}\right)$ denotes the gamma distribution with mean $c_{1}/c_{2}$ and variance $c_{1}/c_{2}^{2}$ to formulate the likelihood. We define the interactions of two exposure variables $X_{ij}$ and $X_{ik}$ for the $i^{th}$ individual as $Z_{i_{jk}}=X_{ij}X_{ik}$ and $Z_{i}={\left(Z_{i_{11}},Z_{i_{12}},\cdots ,Z_{i_{p(p-1)/2}}\right)}^{\prime }$. Hence, $\xi_{i}\left|\lambda_{i}\sim \text{N}\left(U_{i}^{\prime }\alpha +X_{i}^{\prime }\beta +Z_{i}^{\prime }\gamma ,\frac{1}{\lambda_{i}}\right)\right.$ and $\lambda_{i}\sim \text{G}\left(\frac{v}{2},\frac{v}{2}\right)$, where $\beta =\left(\beta_{1},\beta_{2},\ldots ,\beta_{p}\right)$ and $\gamma =\left(\gamma_{11},\gamma_{12},\ldots ,\gamma_{p(p-1)/2}\right)$. Hence, the complete data likelihood is as follows:

(4) $$\begin{array}{ll} \pi \left(Y|X\right) & \;=\prod_{i=1}^{N}\left[Y_{i}1\left\{\xi_{i}>0\right\}+\left(1-Y_{i}\right)1\left\{\xi_{i}<=0\right\}\right]\\ \; & \;\times {\left(2\pi \right)}^{-\frac{1}{2}}\lambda_{i}^{\frac{1}{2}}exp\left(-\frac{\lambda_{i}}{2}{\left(\xi_{i}-U_{i}^{'}\alpha -X_{i}^{'}\beta -Z_{i}^{'}\gamma \right)}^{2}\right)\\ \; & \;\times \frac{{\left(\frac{v}{2}\right)}^{\frac{v}{2}}}{\Gamma \left(\frac{v}{2}\right)}\lambda_{i}^{\frac{v}{2}-1}exp\left(-\frac{\lambda_{i}v}{2}\right). \end{array}$$

The main and interaction effects can be estimated by choosing a vague prior such that $\beta_{j},\gamma_{jk}\sim \text{N}\left(0,10^{2}\right)$; this is approximately a maximum likelihood approach. Incorporating a global–local shrinkage parameter might be a good option as it gathers information from the data to determine the amount of shrinkage that needs to be incorporated. To that end,

(5) $$\beta_{j}\sim \text{N}\left(0,\frac{1}{a\eta_{j}}\right),\;\gamma_{jk}\sim \text{N}\left(0,\frac{1}{b\theta_{jk}}\right).$$

The shrinkage priors mentioned in Eq. (5) do not imply the hierarchical principle [14, 15], where interactions are only considered when corresponding main effects are present. Recent work [1] considered including this hierarchical condition by incorporating the following prior distribution:

(6) $$\begin{array}{ll} & \beta_{j}\sim \text{N}\left(0,\frac{1}{a\eta_{j}}\right),\gamma_{jk}\sim \text{N}\left(0,\frac{1}{b\eta_{j}\eta_{k}\theta_{jk}}\right),\\ & \eta_{j}\sim \text{G}(1,1),\theta_{jk}\sim \text{G}(1,1). \end{array}$$

The prior distribution in Eq. (6) follows the global–local prior specification of [16]. In this formulation, the local shrinkage parameter controls the degree of shrinkage for each individual and the global shrinkage parameter controls the overall shrinkage. Here for the main effect regression coefficient $\beta_{j}$, we consider a predictor-specific local shrinkage parameter $\eta_{j}$ that controls the deviation in the degree of shrinkage for each exposure variable and a global parameter a that controls the overall shrinkage of the main effects towards the origin. Similarly, for the interaction effect regression coefficient $\gamma_{jk}$, the predictor-specific local shrinkage parameter for each interaction term $\theta_{jk}$ controls the degree of shrinkage for each interaction term, while the global shrinkage parameter b controls the overall shrinkage. We define, $\eta ={\left(\eta_{1},\eta_{2},\ldots ,\eta_{p}\right)}^{\prime }$, i.e., p-dimensional vector of local shrinkageparameters of main effect and $\theta ={\left(\theta_{1},\theta_{2},\ldots ,\theta_{p(p-1)/2}\right)}^{\prime }$ the local shrinkage parameters for interaction effects. As a prior choice for both $\eta_{j}$’s and $\theta_{jk}$’s, we consider a heavy tail distribution G(1, 1) distribution with mean and variance 1 to avoid overshrinking issues and incorporate variability. The larger values of $\eta_{j}$’s and $\theta_{jk}$’s will induce more shrinkage towards zero for the corresponding main effects and interaction effects, respectively, while smaller values indicate less shrinkage to zero. For the global shrinkage parameters a and b, we consider G(1, 1) distribution as a prior choice to incorporate substantial mass near the origin. Finally, we considered a vague prior for $\alpha \sim \text{MVN}\left(0,10^{2}I_{q}\right)$, where $I_{q}$ defines $q^{\text{th}}$-order identity matrix.

The main objective of the shared shrinkage model is to incorporate a link between the main effects and the interaction effects. To that end, Kundu et al. [1] share the information between the $j^{\text{th}}$ main effect and the $(j,k{)}^{\text{th}}$ interaction effects through the local parameters $\eta_{j}$ and $\eta_{k}$. We control the prior variance of $\gamma_{jk}$ by the term $\eta_{j}\eta_{k}$, such that $\gamma_{jk}$ will shrink to zero if at least one of the corresponding main effects $\beta_{j}$ or $\beta_{k}$ is small, i.e., their corresponding local shrinkage parameters $\eta_{j}$ or $\eta_{k}$ is large or the local shrinkage parameter of the interaction term $\theta_{jk}$ is large itself. Similarly, if the main effects are sizeable, i.e., their corresponding $\eta_{j}$’s and $\eta_{k}$’s are small, that will induce less shrinkage for the corresponding interaction term $\gamma_{jk}$.

**Fig. 4:**
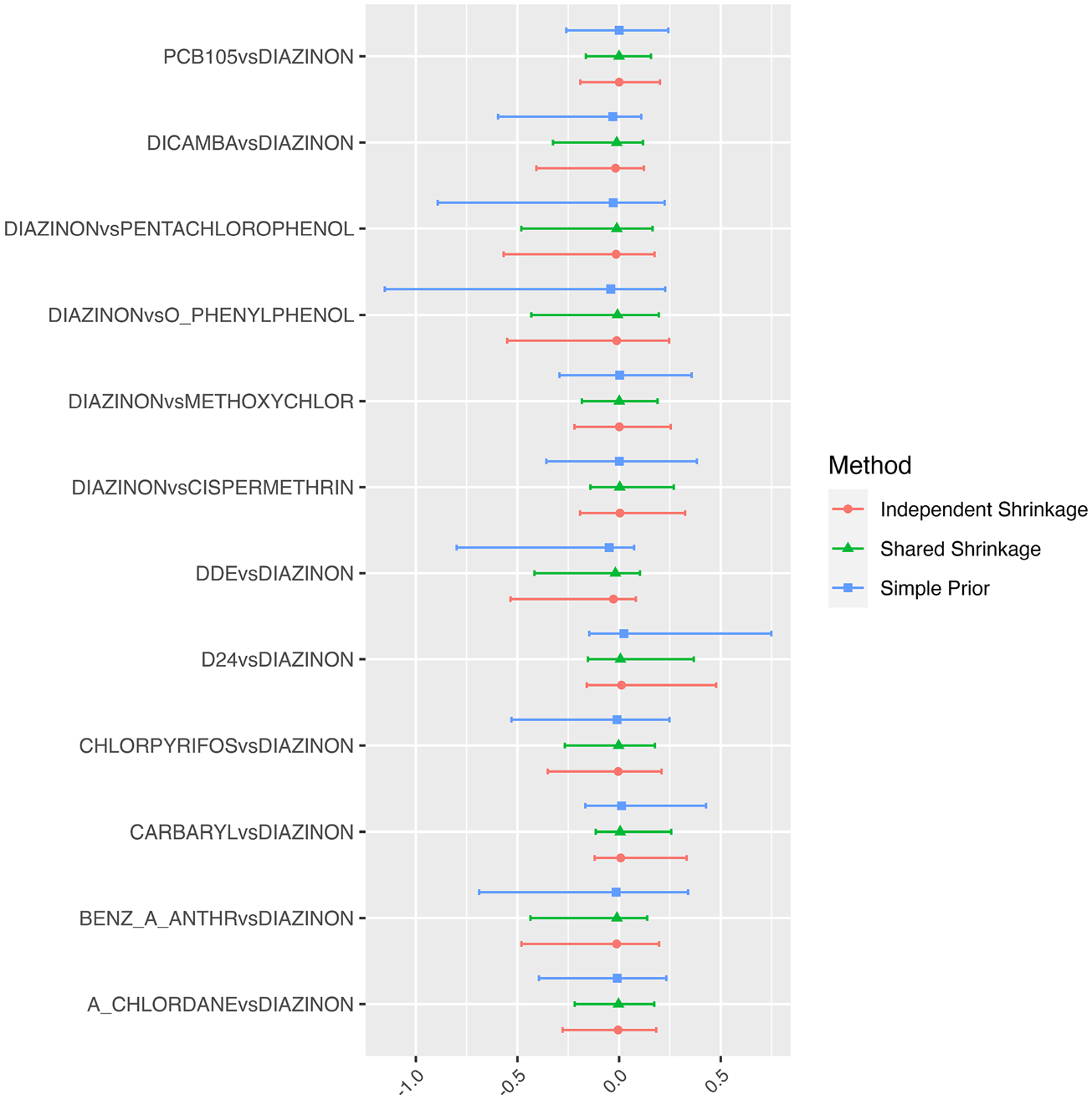
Comparison of Interaction effects with Diazinon with different model

Figure 4 shows a comparison of estimated interactions for the NHL study. For all interaction terms on the three models, the 95% HPD interval for $\gamma_{jk}$ contains zero, suggesting that there is no evidence for any two-way interaction among the components of the mixture. The order of the magnitude of the interval lengths from largest to smallest is the vague, independent shrinkage, and the shared shrinkage prior, respectively, demonstrating the efficiency advantages of incorporating a shrinkage prior along with exploiting the hierarchical assumption into parameter estimation.

**Fig. 5:**
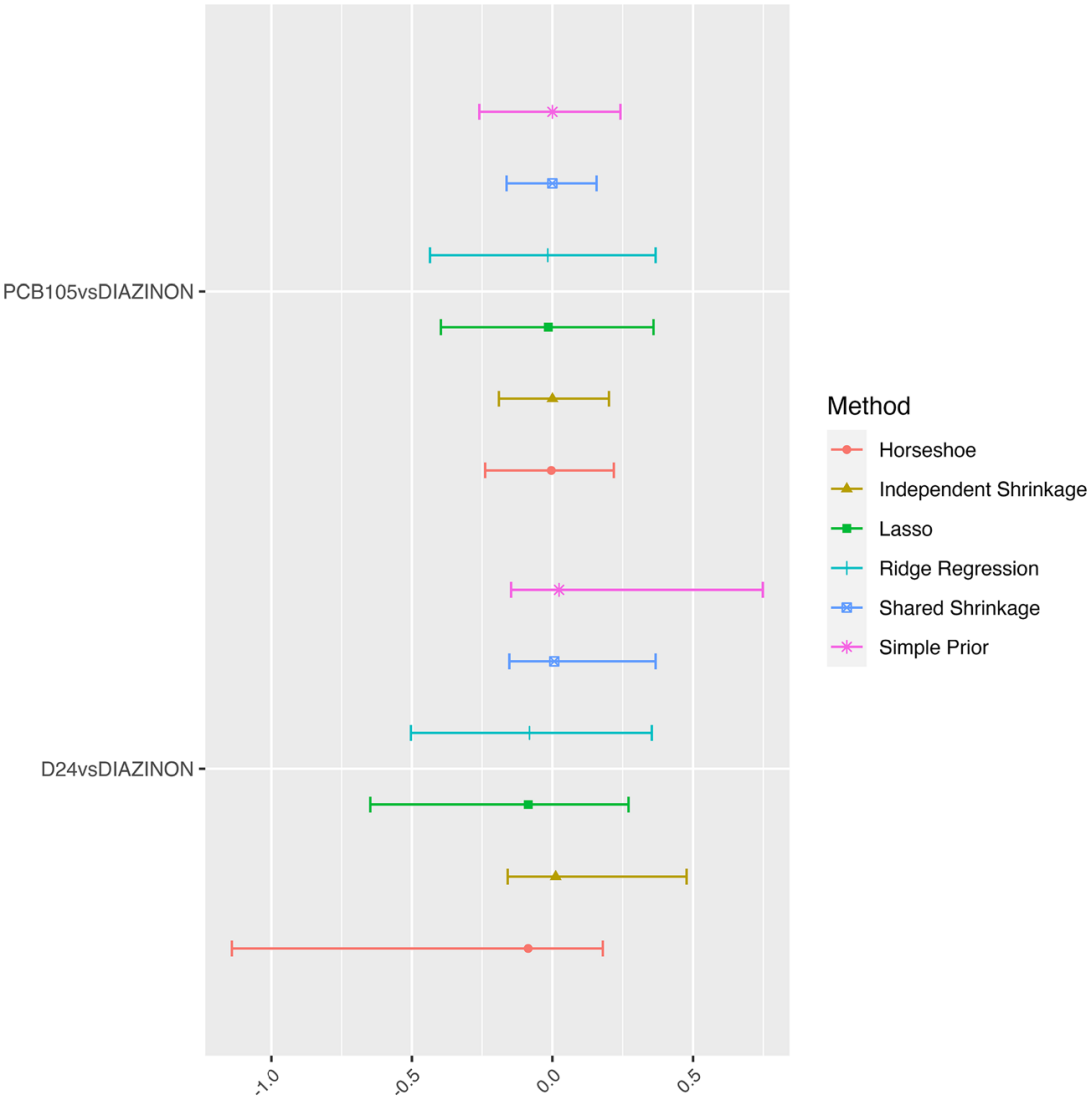
Comparison of Interaction effects with Diazinon from with different prior choices

As an additional sensitivity analysis, we also examined other shrinkage priors including a ridge [17], Lasso [18], and horseshoe [19] prior. Under a linear exposure (each exposure contributes a single linear term) model, estimation with these shrinkage priors can be done directly with the R package *bayesreg*. However, we emphasize that these methods do not incorporate any hierarchical structure. Figure 5 shows the comparison of interaction estimation using different shrinkage priors. As we have 91 interaction terms and are comparing six different methods, we illustrate the results with the estimation of two interaction terms. Although all intervals obtained with all six methods contain zero, the shared shrinkage prior approach has the narrowest interval, and is therefore most efficient.

So far, we described the modeling of a linear exposure and outcome relationship. In practice, exposure may be non-linear requiring more than one regression parameter for each exposure. Kundu et al. [1] extended their methodology to capture those non-linear exposure-outcome relationships using the following logistic regression model:

(7) $$\text{logit}P\left(Y_{i}=1|X_{ij},U_{i}\right)=U_{i}^{'}\alpha +\overset{p}{\underset{j=1}{\sum }}g_{j}\left(X_{ij}\right)+\overset{p}{\underset{j=1}{\sum }}\overset{p-1}{\underset{k=j+1}{\sum }}f_{jk}\left(X_{ij},X_{ik}\right).$$

We use a polynomial representation to model the non-linear exposure effect of each chemical. These polynomial effects are incorporated in the main and interaction effects by using Eq. (7) with functions $g_{j}\left(X_{ij}\right)=X_{ij}^{\prime }\beta_{j}$ and $f_{jk}\left(X_{ij},X_{ik}\right)=Z_{jk}^{\prime }\gamma_{jk}$ and the following logistic regression:

(8) $$\text{logit}P\left(Y_{i}=1|X_{i},Z_{i},U_{i}\right)=U_{i}^{'}\alpha +\overset{p}{\underset{j=1}{\sum }}X_{ij}^{'}\beta_{j}+\overset{p}{\underset{j=1}{\sum }}\overset{p-1}{\underset{k=j+1}{\sum }}Z_{jk}^{'}\gamma_{jk},\;i=1,2,\ldots ,N,$$

where $X_{ij}={\left(X_{ij},X_{ij}^{2}\right)}^{\prime }$ and $Z_{jk}={\left(X_{ij}X_{ik},X_{ij}^{2}X_{ik},X_{ij}X_{ik}^{2},X_{ij}^{2}X_{ik}^{2}\right)}^{\prime }$ and the regression coefficients $\beta_{j}={\left(\beta_{j1},\beta_{j2}\right)}^{\prime }$ and $\gamma_{jk}={\left(\gamma_{jk1},\gamma_{jk2},\gamma_{jk3},\gamma_{jk4}\right)}^{\prime }$.

Most interesting for our application is to incorporate exposures that are subject to LOD in a robust manner. We incorporate a two-parameter per exposure model that was recently discussed for univariate exposure relationships Chiou et al. [20] and Ortega-Villa et al. [21]. In this formulation, (i) the first component indicates whether the exposure for a single chemical is above the detection limit and ii) the second part shows the value of the exposure effect if it is above the detection limit. This parameterization allows a flexible modeling approach in spite of treating lower LOD as left censored. Hence, Kundu et al. [1] represent the extension of their work using Eq. (7) as follows:

(9) $$\begin{array}{rr} g_{j}\left(X_{ij}\right) & \;=\beta_{j1}I\left(X_{ij}\geq C_{j}\right)+\beta_{j2}I\left(X_{ij}\geq C_{j}\right)\left(X_{ij}-C_{j}\right)\\ f_{jk}\left(X_{ij},X_{ik}\right) & \;=\gamma_{jk1}I\left(X_{ij}\geq C_{j}\right)I\left(X_{ik}\geq C_{k}\right)+\gamma_{jk2}\left(X_{ij}-C_{j}\right)I\left(X_{ij}\geq C_{j}\right)I\left(X_{ik}\geq C_{k}\right)\\ & \;+\gamma_{jk3}\left(X_{ik}-C_{k}\right)I\left(X_{ij}\geq C_{j}\right)I\left(X_{ik}\geq C_{k}\right)\\ & \;+\gamma_{jk4}\left(X_{ij}-C_{j}\right)\left(X_{ij}-C_{k}\right)I\left(X_{ij}\geq C_{j}\right)I\left(X_{ik}\geq C_{k}\right), \end{array}$$

where $\beta_{j1}$ defines the log odds of disease at the value of the detection limit relative to the log odds of disease below the detection limit, $\beta_{j2}$ defines the log odds ratio of disease for a one-unit change in exposure above the detection limit. The interactive effects are measured using the parameter vector $\gamma_{jk}$. Here, $\gamma_{jk1}$ represents the interactive effect when both the $j^{th}$ and $k^{th}$ chemicals are above the detection limit, $\gamma_{jk4}$ represents the interactive effect of increasing $X_{ij}$ and/or $X_{ik}$ when both markers are above the detection limit, and $\gamma_{jk2},\;\gamma_{jk3}$ are cross-product interaction effects.

**Fig. 6:**
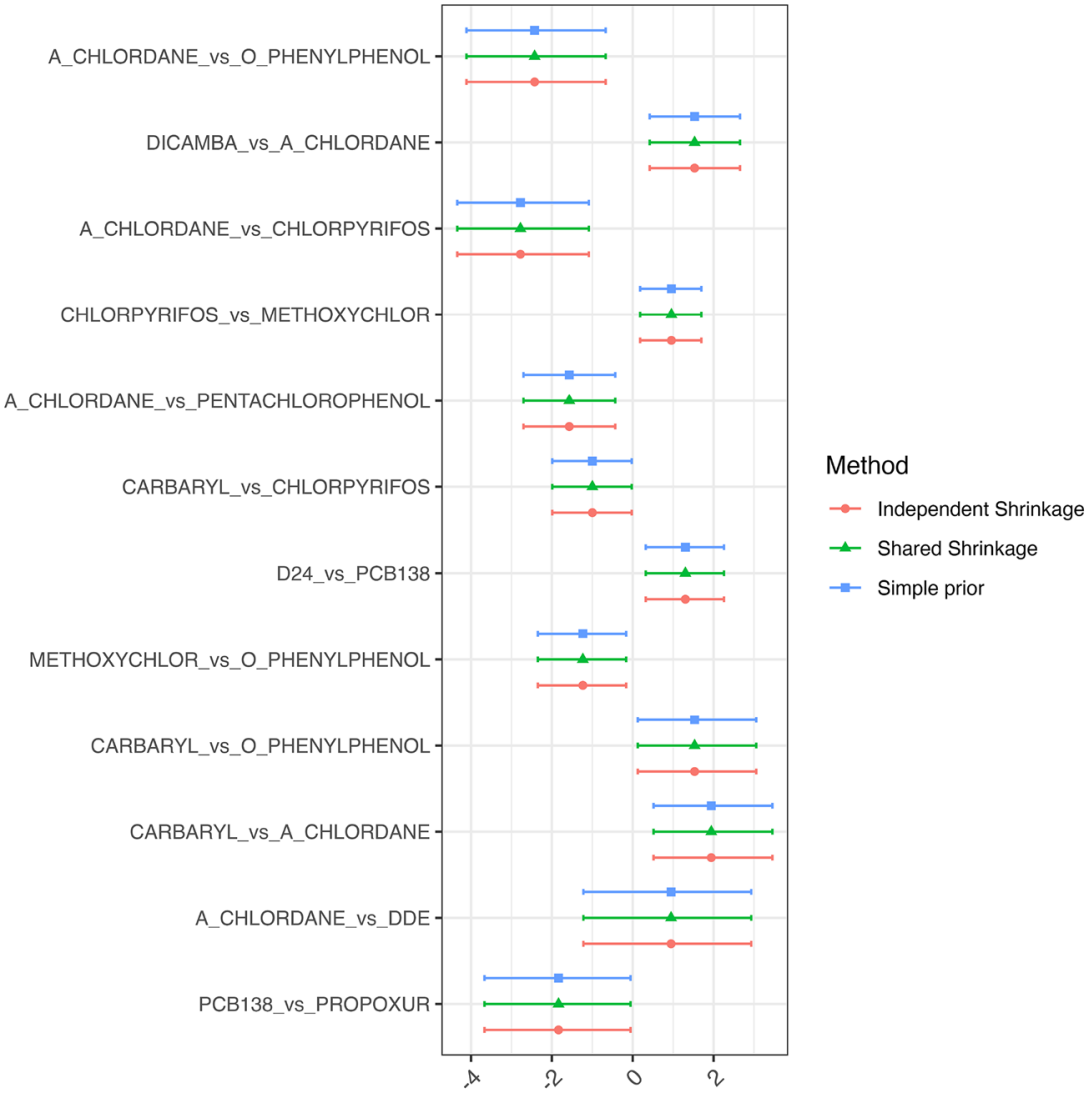
Comparisons between randomly chosen slope vs slope $\left(\gamma_{jk4}\right)$ interaction effects

Using two parameters per exposure model, Fig. 6 shows that we found multiple interaction effects, some of which demonstrated positive synergy between chemicals and others showing a negative interaction. The fact that the results are different as compared with the imputation approach suggests that imputing based on a parametric normal model may be problematic.

## 5 A Latent Functional Approach

To incorporate non-linear exposure risk relationships in a binary regression setting, Kim et al. [5] proposed the latent functions approach, where the individual effects for each exposure in a risk model can be written as the sum of unobserved functions. They showed that the relationship between chemical exposures and risk becomes more flexible as the number of latent functions increases, and complex exposure relationships can represented with only a few such functions. In this article, we extend the methodology to allow for a separate set of latent classes for the main and interaction effects, respectively.

As in Sect. 4, let $Y_{i}$ be a binary outcome for the $i^{\text{th}}$ individual. Also, let $U_{i}={\left(U_{i1},\ldots ,U_{iq}\right)}^{\prime }$ denote a q-dimensional vector of covariates for the $i^{th}$ individual and $\alpha ={\left(\alpha_{1},\ldots ,\alpha_{q}\right)}^{\prime }$ denote the vector of regression coefficients corresponding to the q covariates. Furthermore, let $X_{ij}$ for main effects denote the chemical exposure for the $j^{th}$ chemical on the $i^{th}$ individual, j=1,…,p, and $Z_{ik}=X_{ij_{1}}X_{ij_{2}}$ for two-way interactions, $j_{2}=j_{1}+1,\ldots ,p,\;j_{1}=1,\ldots ,p,\;k=1,\ldots ,K$ with K=p(p-1)/2, and i=1,…,N. Similar to Kim et al. [5], we use a binary regression model with interactions based on a finite number of non-linear functions using latent variable approach of Albert and Chib [11] as follows:

(10) $$\begin{array}{l} Y_{i}=\{\begin{array}{ll} 1 & \text{if }\xi_{i}\geq 0\\ 0 & \text{if }\xi_{i}<0 \end{array}\text{ and}\\ \xi_{i}=U_{i}^{'}\alpha +\overset{p}{\underset{j=1}{\sum }}\overset{L}{\underset{l=1}{\sum }}1\left(g_{j}=l\right)f_{l}\left(X_{ij}\right)+\overset{K}{\underset{k=1}{\sum }}\overset{M}{\underset{m=1}{\sum }}1\left(h_{k}=m\right)s_{m}\left(Z_{ik}\right)+\epsilon_{i}, \end{array}$$

where $f_{l}\left(X_{ij}\right)$ is a functional form of $X_{ij}$ for the $l^{\text{th}}$ latent class, $g_{j}$ is a latent membership indicator with $P\left(g_{j}=l\right)=\omega_{l},\;L$ is a fixed number of latent classes (1≤L≤p), $s_{m}\left(Z_{ik}\right)$ is a functional form of $Z_{ik}$ for the $m^{\text{th}}$ latent class, $h_{k}$ is a latent membership indicator with $P\left(h_{k}=m\right)=\Psi_{m},\;M$ is a fixed number of latent classes (m≤M≤K), and $\epsilon_{i}$ follows a t-distribution with indexed by the degrees of freedom v=7. Note that the indicator function 1{A} is defined as 1{A}=1 if A is true and 0 otherwise. In this paper, we assume a polynomial regression function of order c to capture the non-linear structure for $f_{l}\left(X_{ij}\right)$ and $s_{m}\left(Z_{ik}\right)$ in Eq. (10) as $f_{l}\left(X_{ij}\right)=\beta_{l1}X_{ij}+\cdots +\beta_{lc}X_{ij}^{c}\equiv X_{ij}^{*\prime }\beta_{l}$ and $s_{m}\left(Z_{ik}\right)=\gamma_{m1}Z_{ik}+\cdots +\gamma_{mc}Z_{ik}^{c}\equiv Z_{ik}^{*\prime }\gamma_{m}$, where $X_{ij}^{*}={\left(X_{ij},\ldots ,X_{ij}^{c}\right)}^{\prime },\;\beta_{l}={\left(\beta_{l1},\ldots ,\beta_{lc}\right)}^{\prime },\;Z_{ik}^{*}={\left(Z_{ik},\ldots ,Z_{ik}^{c}\right)}^{\prime },\;$ and $\gamma_{m}={\left(\gamma_{m1},\ldots ,\gamma_{mc}\right)}^{\prime }$. The latent variable $\xi_{i}$ in Eq. (10) can be rewritten as

(11) $$\begin{array}{ll} \xi_{i} & \;=U_{i}^{\text{'}}\alpha +\overset{p}{\underset{j=1}{\sum }}\overset{L}{\underset{l=1}{\sum }}1\left(g_{j}=l\right)X_{ij}^{\text{*'}}\beta_{l}+\overset{K}{\underset{k=1}{\sum }}\overset{M}{\underset{m=1}{\sum }}1\left(h_{k}=m\right)Z_{ik}^{\text{*'}}\gamma_{m}+\epsilon_{i}\\ \; & \;=U_{i}^{\text{'}}\alpha +\overset{p}{\underset{j=1}{\sum }}X_{ij}^{\text{*'}}\delta_{j}^{x}+\overset{K}{\underset{k=1}{\sum }}Z_{ij}^{\text{*'}}\delta_{k}^{z}+\epsilon_{i}, \end{array}$$

where $\delta_{j}^{x}=\sum_{l=1}^{L}1\left(g_{j}=l\right)\beta_{l}$ and $\delta_{k}^{z}=\sum_{m=1}^{M}1\left(h_{k}=m\right)\gamma_{m}$, corresponding to regression coefficients for the $j^{\text{th}}$ main effect and the $k^{\text{th}}$ interaction term, respectively. The similar prior distributions and MCMC algorithm in Kim et al. (2023) are used in the analysis. To help obtain numerical stability in the implementation of the MCMC sampling algorithm, we standardized all of covariates by subtracting their sample means and then dividing by their sample SDs. All variables for main effects and interactions are standardized by dividing by its maximum value.

We assumed a cubic polynomial regression function for $f_{l}\left(x_{ij}\right)$ and $s_{m}\left(Z_{ik}\right)$ in Eq. (10) to incorporate a flexible functional form (c=3) in this paper. We considered models with various L and M to choose the number of latent classes to characterize the simultaneous effects of all chemicals on cancer risk. Table 1 shows the estimated posterior probabilities for $\omega_{l}$ and $\Psi_{m}$ for the model with L=5 and M=5, demonstrating that the posterior probabilities of $\omega_{l}$ and $\Psi_{m}$ for L>3 and M>3 were almost zero, suggesting many latent profiles are not needed.

**Fig. 7:**
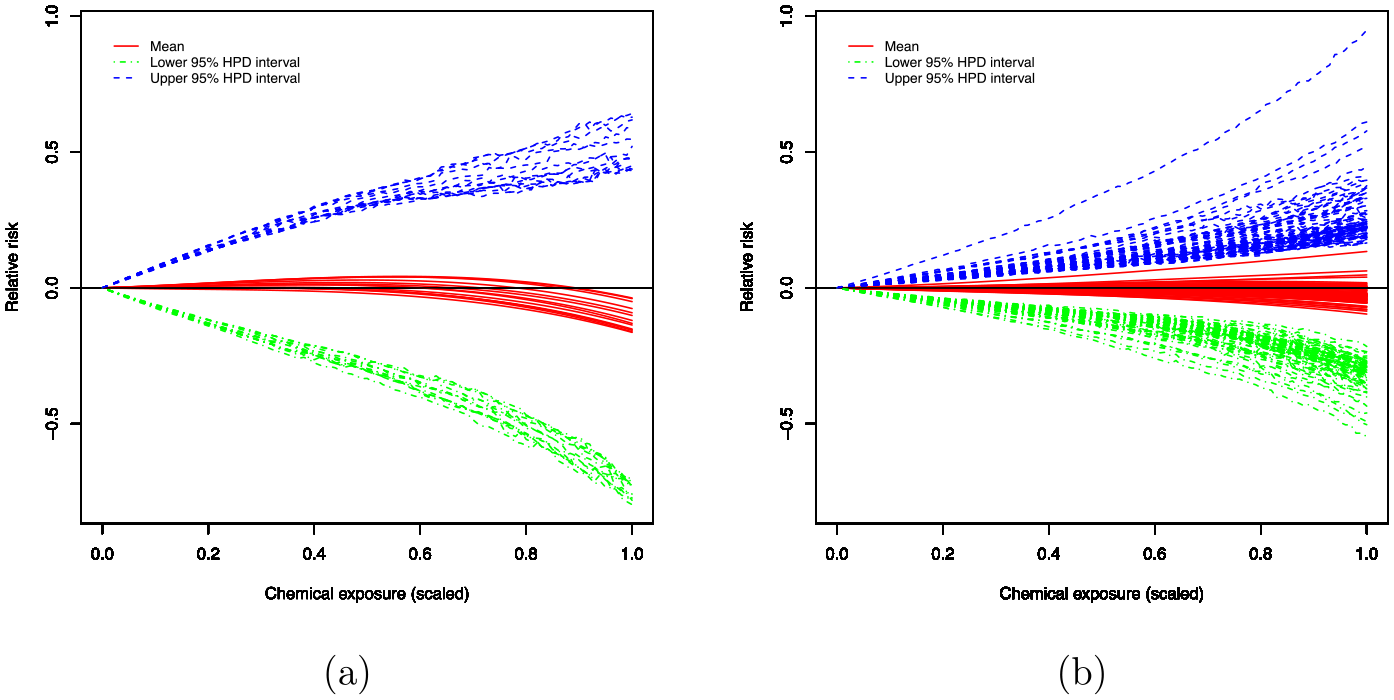
Plots of the estimated log relative risks (relative to no exposure) as a function of chemical exposure with the posterior mean and 95% HPD intervals under the model with L=5 and M=5: **a** main effects; **b** interaction effects

Figure 7 shows the estimated log relative risks for the individual functional relationships and the corresponding 95% HPD intervals for 14 main effects and 91 interaction terms under the model with L=5 and M=5, respectively, showing that 95% HPD intervals include zero line and none of the main effects and interaction terms have relationship with NHL.

## 6 Discussion

Recently, there have numerous statistical approaches proposed in the statistics literature for studying the interactions among chemical mixture components. These approaches perform well under a correct model specification. However, there have been few comparisons of these methodologies on actual study data. This paper compares numerous recently developed approaches to a case–control study of NHL that examined the effects of multiple pollutants on cancer risk.

A challenging analytic issue in the analysis was the high proportion of LOD among chemicals. The original analyses of the study [9] used a simple imputation method for imputing values below the LOD. Using these imputations, we found that all the methods showed similar interaction effect estimates that were consistent with zero. Although we only used one realization from the imputation model for all analyses, we obtained nearly identical estimations using other realizations (data not shown).

Recognizing that the imputation approaches make strong assumptions on the distributions below the LOD, we conducted an additional analysis where each chemical exposure was represented by two parameters; one parameter for being above the LOD and the second for the slope when above this limit. Our two-parameter per exposure model does not require those strong assumptions. These analyses focused on the shrinkage estimation since this class of models can more easily be extended in a flexible way. Many interactions were identified with this formulation. In part, this can be explained if the imputation methods, which are difficult to validate, are inadequate (see Ortega-Villa et al. [21] for a simulation study with one exposure measurement). These results motivate the future methodological extending approaches such as BKMR and the latent functional approach to more flexibly incorporate LOD.

The different methods had different assumptions about the linearity of the exposure effects. BKMR introduces flexible relationships by the specification of the kernel function. However, it is not totally transparent what explicit assumptions are made on the linearity by specifying a particular kernel function. The latent functions approach explicitly assumes a polynomial assumption on the exposure relationships.

Each of the proposed methods used the scaled absolute exposure values in the analyses. We also applied all the methods to percentiles of the exposure values rather than the absolute measurements. We were able to fit all methods with the exception of BKMR for these transformed exposure values. We were unable to come up with a reason for the computational failure of BKMR in this situation. However, for all other methods, we obtained similar inferences to those obtained with the absolute values (data not shown).

The methodology comparison focused on analyses from a case–control study. All the methods except BKMR have a direct relative-risk interpretation since we incorporated a logit link function and the NHL is a rare disease. The interpretation for BKMR is less clear since this approach uses a probit link function to relate the mixture components to cancer risk. The methodology and comparisons naturally apply to cohort studies with binary outcomes. Extensions to survival and longitudinal outcomes are areas for future research.

## Figures and Tables

**Table 1:** Posterior probability of $\omega_{l}$ and $\Psi_{m}$ for model with L=5 and M=5

L and M	$\omega_{l}$	$\Psi_{m}$
1	0.8988	0.8805
2	0.0954	0.1112
3	0.0055	0.0080
4	0.0002	0.0004
5	< 0.0001	< 0.0001

## Data Availability

Data are available upon request with the required data agreement policy. All codes for the different models are available in the GitHub account.

## Appendix

The name of all chemicals in the dataset are listed below:

1. Pentachlorophenol
2. Propoxur
3. O-phenylphenol
4. Transpermethrin
5. Cispermethrin
6. Methoxychlorene
7. Diazinon
8. DDT
9. DDE
10. Chlorpyrifos
11. G-chlordane
12. A-chlordane
13. Carbaryl
14. PCB 180
15. PCB 170
16. PCB 153
17. PCB 138
18. PCB 105
19. Indeno-Pyr
20. Dibenz-Anthracene
21. Chrysene
22. Benzo-A-Pyrene
23. Benz-k-Fluoranthene
24. Benz-A-Anthracene
25. Dicamba
26. 2,4-D; D24 chemical in the figure indicates the chemical 2,4-D.
